# Immobilization of L-Asparaginase on biofunctionalized magnetic graphene oxide nanocomposite: A promising approach for Enhanced Stability and reusability

**DOI:** 10.1016/j.heliyon.2024.e40072

**Published:** 2024-11-01

**Authors:** Maryam Monajati, Nasim Ariafar, Mehdi Abedi, Sedigheh Borandeh, Ali Mohammad Tamaddon

**Affiliations:** a Department of Pharmaceutical Nanotechnology, School of Pharmacy, Shiraz University of Medical Sciences, Shiraz, Iran; b Center for Nanotechnology in Drug Delivery, Shiraz University of Medical Sciences, Shiraz, Iran; c Student Research Committee, Shiraz University of Medical Sciences, Shiraz, Iran

**Keywords:** Protein, Conjugation, Catalytic activity, Stability, Reusability

## Abstract

The application of the amidohydrolase enzyme, L-asparaginase (ASNase), as a biocatalyst in the food and pharmaceutical industries has garnered significant interest. However, challenges such as hypersensitivity reactions, limited stability, and reusability under various operational conditions have hindered its cost-effective utilization. This paper introduces a novel nano-support for ASNase immobilization, namely the nanocomposite of iron oxide magnetic nanoparticles and amino acid-decorated graphene oxide (GO-Asp-Fe_3_O_4_). Characterization using FTIR spectroscopy, FE-SEM, and TEM microscopy revealed the homogeneous distribution of iron oxide nanoparticles on the surface of GO sheets. The effects of carrier functionalization and carrier-to-protein ratio on the immobilization of ASNase were studied to optimize the immobilization conditions. The magnetized nanocomposite of ASNase exhibited a 4.4-fold lower Michaelis-Menten constant (Km), suggesting an enhanced affinity for the substrate. The immobilized ASNase demonstrated two to eight times higher thermostability compared to the free enzyme and showed an extremely extended pH stability range. Furthermore, the immobilized enzyme retained over 80 % of its initial bioactivity after eight repeated reaction cycles. These findings suggest that the immobilization of ASNase on GO-Asp- Fe_3_O_4_ nanocomposite could be a viable option for industrial applications.

## Graphical abstract

Enhanced Stability and Reusability of Immobilized L-Asparaginase on biofunctionalized Magnetic Graphene Oxide Nanocomposite.

**Figure:**
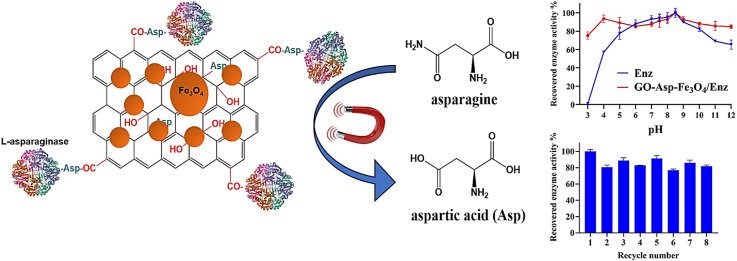

## 1 Introduction

L-asparaginase (ASNase) is of high interest as a biocatalyst in the food and pharmaceutical industry. The amidohydrolase activity of ASNase deprives malignant cells of the essential amino acid L-asparagine, making ASNase a crucial treatment for lymphosarcoma and acute lymphoblastic leukemia [1]. In addition, ASNase has been widely used in the food industry to reduce the formation of carcinogenic acrylamide in starch-rich foods [2]. ASNase can also be integrated into biosensors for monitoring the levels of L-asparagine in the patient's blood serum or different food samples [3]. However, prolonged administration of bacterial ASNase has the potential to induce hypersensitivity reactions and the development of neutralizing antibodies, which can limit its effectiveness and tolerability [4]. Limited stability and reusability under various operational conditions is another obstacle to hamper for cost-effective utilization of ASNase in industrial applications [5]. To address these challenges, enzyme immobilization onto solid supports has emerged as a promising strategy to enhance its stability, reusability, and medical performance [6].

In the past few years, the integration of nanomaterials into enzyme immobilization systems has attracted considerable interest, owing to the unique physicochemical properties nanomaterials offer, including high enzyme loading capacity, tunable surface chemistry, and stabilizing effect [7]. Carbon-based nanomaterials, exclusively graphene oxide (GO), are considered promising candidates for enzyme immobilization, offering advantages such as exponentially high surface area and impressive thermal and mechanical properties [8]. In addition, their potential to introduce specific functional groups enables covalent enzyme immobilization [9]. To date, GO has been employed as a matrix for the immobilization of diverse enzymes such as α-chymotrypsin [10], glucose oxidase [11], glutathione peroxidase [12], lipase [13], and horseradish peroxidase [14]. Nevertheless, the challenges posed by GO toxicity and its limited dispersion stability in biological environments have hindered its widespread utilization in the biomedical field. Hence, surface functionalization of GO with polymers and biomolecules presents a viable solution to enhance its biocompatibility [15]. Previous investigations from our laboratory have demonstrated that the functionalization of GO with L-aspartic acid (L-Asp) could efficiently reduce the cytotoxicity and apoptosis induction compared with various amino acids making it a promising candidate for pharmaceutical applications [16].

Another important challenge is that the immobilized enzyme in these materials is difficult to recover. Magnetic GO has offered the combined advantages of using both GO and magnetic nanoparticles for enzyme immobilization. The incorporation of magnetic nanoparticles into enzyme immobilization platforms can facilitate the separation and recovery of the biocatalyst from reaction mixtures using an external magnetic field [17]. This innovation also contributes to the higher operational stability of enzymes, ensuring sustained catalytic performance across diverse pH levels and temperature ranges [18]. Additionally, the extended surface area of magnetic graphene oxide accommodates a high enzyme load, potentially amplifying catalytic efficiency. These characteristics highlight the material's ability to simplify biotechnological processes, improving the efficiency and cost-effectiveness of using enzymes in industrial applications [19].

**Fig. 1:**
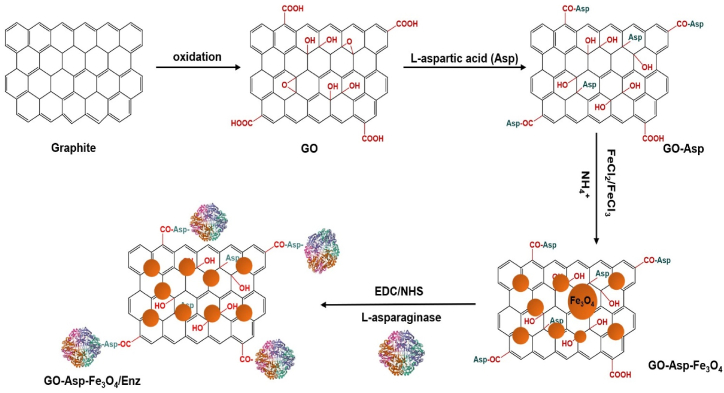
Schematic immobilization of L-asparaginase (Enz) on magnetic graphene oxide functionalized with L-aspartic acid (Asp).

In our previous work, we reported ASNase immobilization on L-Asp decorated GO (GO-Asp) [20]. In addition to providing high biocompatibility, L-Asp was used to prevent structural damage to the enzyme. Despite our promising results in ASNase stability, a strong technological need for improved reusability was desired. Thus, to explore the efficacy of magnetic GO matrix for ASNase immobilization, we initially synthesized GO-Asp, which was subsequently functionalized through an in-situ co-precipitation process involving Fe ions (Fig. 1). In the next steps, the activity, pH and thermal stability, and reusability of covalently immobilized ASNase were investigated under various temperature and pH conditions.

## 2 Experiments

### 2.1 Materials

ASNase (Leunase®) was sourced from Kyowa Hakko Kirin Co. Ltd. Natural graphite powder with a particle size of 70 μm, ferric chloride hexahydrate (98 %), ferrous chloride tetrahydrate, hydrogen peroxide (H_2_O_2_), sulfuric acid (98 %), hydrochloric acid (HCl), potassium permanganate (KMnO_4_), and dimethyl sulfoxide (DMSO) were purchased from Merck Chemical Company. N-hydroxy succinimide (NHS), 2,4,6-trinitrobenzene sulfonic acid (TNBS), and 1-Ethyl-3-(3-dimethylaminopropyl) carbodiimide (EDC), were acquired from Sigma Aldrich Company (USA). The Nessler reagent was obtained from Daejung Company (China).

### 2.2 Instruments

The Fourier transform infrared (FT-IR) spectra were acquired utilizing a vertex 70 spectrophotometer from Bruker in Germany. The measurements were conducted at a resolution of 4 cm^−1^, employing KBr pellets for the solid samples. Raman spectroscopy was performed using a Tekram P50C0R10 Raman Spectrometer, equipped with a Nd:YAG laser source operating at a wavelength of 532 nm. Field emission scanning electron microscopy (FE-SEM) analysis was performed with a HITACHI S-4160 SEM from Japan. For transmission electron microscopy (TEM), a Philips CM 120 operated in the Netherlands at a voltage of 150 kV was used. The determination of hydrodynamic size and surface charge involved the application of dynamic light scattering spectrometry from Microtrac® in the USA and Zetasizer Nanoflex®.

### 2.3 Synthesis of GO-Asp-Fe_3_O_4_ nanoparticles

First, GO-Asp was prepared according to our previously reported method [20]. Briefly, 100 mg GO, prepared through the Hummers' method [21], was mixed with 300 mg L-Asp in 20 mL of NaOH (0.2 M) and stirred for 72 h at ambient temperature. The product was separated using centrifugation (2700 g, 10 min) and washed several times with water and ethanol. Thereafter, to expose carboxylic acid groups on the GO surface, the GO-Asp was ultrasonicated in HCl 5 % and dried under vacuum. GO-Asp-Fe_3_O_4_ was then prepared by in-situ co-precipitation of Fe ions [22]. In a typical experiment, 50 mg GO-Asp was ultrasonicated in 50 mL of deionized water for 30 min, transferred to a two-neck flask, and heated to 50 °C. Next, 500 mg FeCl_2_·4H_2_O and 250 mg FeCl₃·6H₂Owas gradually added to the reaction mixture under a nitrogen atmosphere with continuous stirring. The pH of the mixture was adjusted in the range of 10–11 using ammonia water (25 % w/v). After 30 min, the black-colored GO-Asp-Fe_3_O_4_ was separated by neodymium magnet decantation (1 T), washed several times with deionized water, and then freeze-dried.

### 2.4 Activation of GO-Asp-Fe_3_O_4_ with EDC

A mixture of GO-Asp-Fe_3_O_4_ (30 mg), EDC (40 mg), and NHS (40 mg) was dispersed in 2 mL phosphate buffer (0.01 M, pH = 7.5). The dispersion was then incubated at 37 °C for 2 h. The activated GO-Asp-Fe_3_O_4_ (for chemical immobilization) or the unmodified GO-Asp-Fe_3_O_4_ (for physical immobilization) were dispersed in borate buffer (0.05 M) to obtain the concentration from 2.5 to 20 mg/mL. The reaction media pH was adjusted to 8.0 for GO-Asp-Fe_3_O_4_/NHS and 8.5 for unmodified GO-Asp-Fe_3_O_4_. ASNase solution (1 mg/mL) was then added to each dispersion and incubated for 2 h at room temperature followed by 24 h at 4 °C. Unreacted ASNase in the immobilization medium was separated using centrifugation at 700*g* for 10 min.

### 2.5 Determination of immobilization efficacy

The extent of immobilization of ASNase on GO-Asp-Fe_3_O_4_ and GO-Asp-Fe_3_O_4_/NHS composites was estimated by calculating the difference between the initial (C_i_) and the unreacted enzyme concentration in the supernatant (C_s_), using the Bradford protein assay. The Bradford reagent was applied to quantify the protein concentration at λ = 595 nm from the calibration curve plotted for standard solutions after correcting their absorbances from the blank absorbance. The following equation was used to calculate the immobilization efficiency percent for triplicate samples:

$$\%\;\text{immobilization}\;\text{efficacy}=\frac{C_{i}-C_{s}}{C_{i}}\times 100$$

### 2.6 Determination of enzyme activity

The Nessler assay was employed to determine the activity of free or immobilized ASNase, and the substrate utilized was L-asparagine. The reaction mixture was prepared by mixing 250 μL L-asparagine (40 mM in 0.05 M borate buffer, pH 8.5), 25 μL of enzyme solution (0.1 mg/mL), and 750 μL borate buffer (0.05 M, pH 8.5) in a microtube. The enzymatic reaction was stopped after 10 min by the addition of 250 μL of 1.5 M trichloroacetic acid. The activity of the ASNase was determined by measuring the liberated ammonia at λ = 480 nm according to a reference curve using ammonium sulfate as a standard. The enzyme activity unit was the amount of enzyme that produced 1 μmol of ammonia in 1 min at 37 °C.

### 2.7 Determination of enzyme kinetic constants

The enzyme kinetic constants for both free and immobilized ASNase were measured by determining the enzyme's activity at various concentrations of L-asparagine (ranging from 0.05 to 15 mM) in a pH 7.4 solution at 37 °C. The data were then analyzed with GraphPad PRISM® 6.0 software using nonlinear regression analysis. From this analysis, the Michaelis constant (Km) and the apparent maximum velocity (Vmax) were determined using the Michaelis-Menten equation: V= (Vmaxּּ·S) ⁄ (Km + S), where V is the corresponding reaction rate and S represents the concentrations of L-asparagine.

### 2.8 Dispersibility

To examine the dispersibility of the GO samples, their aqueous dispersions (1 mg/mL) were obtained via probe sonication. Then, the prepared suspensions were centrifuged for 5 min at 1000 rpm. The absorbance of the supernatants was measured before (A_0_) and following (A_1_) centrifugation with a UV–Vis–NIR spectrophotometer at a wavelength of 808 nm. The percentage of dispersibility for the samples was determined using the equation below:

$$\text{Dispersibility}\;\left(\%\right)=\frac{A_{1}}{A_{0}}\;\times \;100$$

### 2.9 pH and thermal stability

To assess the pH stability, enzyme solutions were incubated at 37 °C for 1 h while maintaining a range of pH values from 3 to 12. The buffers used for this purpose were 0.05 M acetate buffer for pH 3–5, 0.05 M phosphate buffer for pH 6–8, and 0.05 M Tris-HCl for pH 8.5–12. The relative activities of the enzymes were then determined by taking the maximum mean enzyme activity as 100 %. To investigate the effect of temperature, the enzyme activity of both free and immobilized ASNase was measured after 30- and 60-min incubation periods at different temperatures (40, 50, 60, and 70 °C) at pH 8.5 (0.05 M Tris-HCl buffer). The tests were conducted three times.

### 2.10 Reusability

The residual activity of the immobilized enzyme was measured over eight consecutive experiments under its optimum reaction condition. In each cycle, the immobilized enzyme was exposed to L-asparagine as a substrate for 10 min at 37 °C. Subsequently, the enzyme was extracted from the reaction mixture using a magnetic field, washed with 0.05 M borate buffer (pH 8.6), and subsequently introduced into a fresh asparagine solution. The recovered enzyme activity calculated at the end of the first cycle was considered 100 %.

### 2.11 Statistics

The quantitative data obtained from the experiments were expressed as mean ± standard deviation (SD). Statistical analysis was performed using Prism Software (GraphPad, USA). One-way analysis of variance (ANOVA) was used to compare the mean values among multiple groups, with statistical significance set at p < 0.05.

## 3 Results and discussion

### 3.1 Synthesis of GO-Asp-Fe_3_O_4_ nanoparticles

**Fig. 2:**
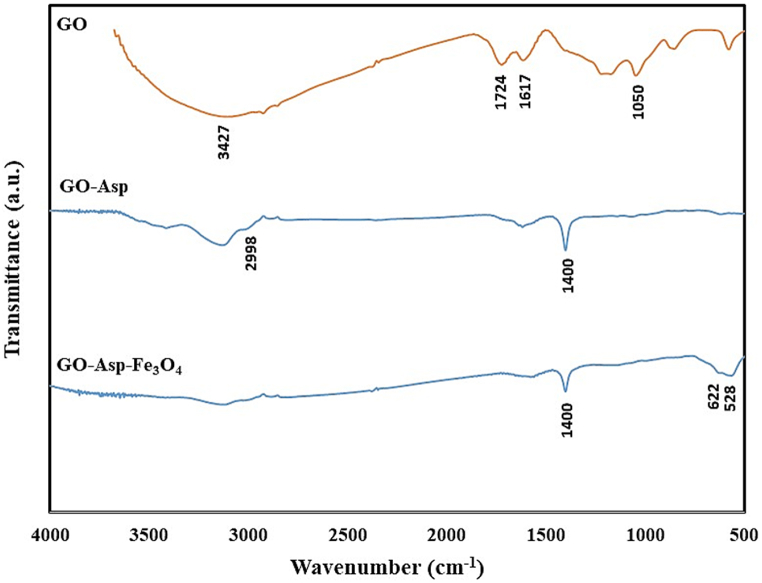
FT-IR absorption spectra of GO, GO-Asp, and GO-Asp-Fe_3_O_4_.

The synthesis of GO-Asp-Fe_3_O_4_ was investigated using FT-IR, XRD, and VSM. The FT-IR spectrum of GO (Fig. 2) revealed characteristic peaks at 3427 cm^−1^, 1724 cm^−1^, 1617 cm^−1^, and 1050 cm^−1^ that could be assigned to the O–H, the C=O carboxylic group, the C=C and C–O stretching vibrations. After treatment with L-Asp, the intensity of these peaks decreased. The FT-IR spectrum of GO-Asp also displays two peaks at 1622 cm^−1^ and 1400 cm^−1^, which correspond to the amide carbonyl group and C-O stretching of the carboxylic acid group, respectively. Moreover, the peak at 2998 cm^−1^ corresponds to the alkane group of aspartic acid, which confirms the functionalization of the GO nanosheets. Similar bands are also observed in the case of GO-Asp- Fe_3_O_4_. In addition, the appearance of two peaks at 559 cm^−1^ and 630 cm^−1^ suggests the presence of the Fe-O bond, which corresponds to the existence of Fe_3_O_4_ nanoparticles on the graphene oxide surface.

**Fig. 3:**
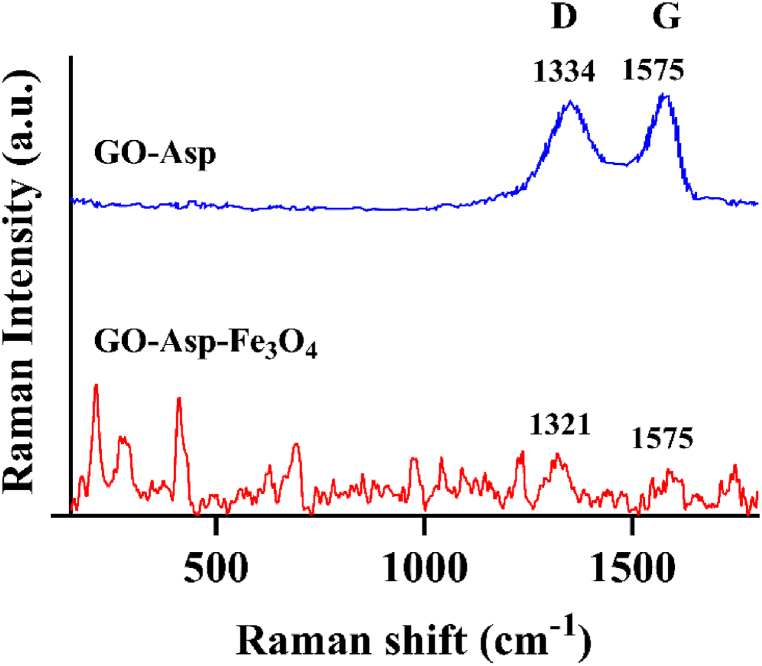
Raman spectra of GO-Asp and GO-Asp-Fe_3_O_4_.

The Raman spectra of GO-Asp and GO-Asp-Fe_3_O_4_ are shown in Fig. 3. For GO-Asp nanoparticles, distinct peaks at 1334 and 1575 cm⁻^1^, corresponding to the D and G bands, were observed. The high ID/IG ratio (≈1.32) suggests a significant presence of structural defects on the GO surface and a partial restoration of the hexagonal carbon lattice with defects. The Raman spectrum of GO-Asp-Fe_3_O_4_ nanoparticles displays several peaks that align with findings in the literature [23,24]. Specifically, peaks at 271, 410, 487, and 571 cm⁻^1^ are attributed to the characteristic vibration modes of Fe-O bonds [25,26]. In the Raman spectra of the magnetized particles, the intensities of the G and D bands are notably reduced. This decrease is likely due to the dilution of GO sheets by incorporating Fe_3_O_4_ nanoparticles, resulting in relatively lower band intensities. Additionally, the attenuation of these peaks may indicate charge transfer from Fe_3_O_4_, a phenomenon commonly observed in such systems [24].

**Fig. 4:**
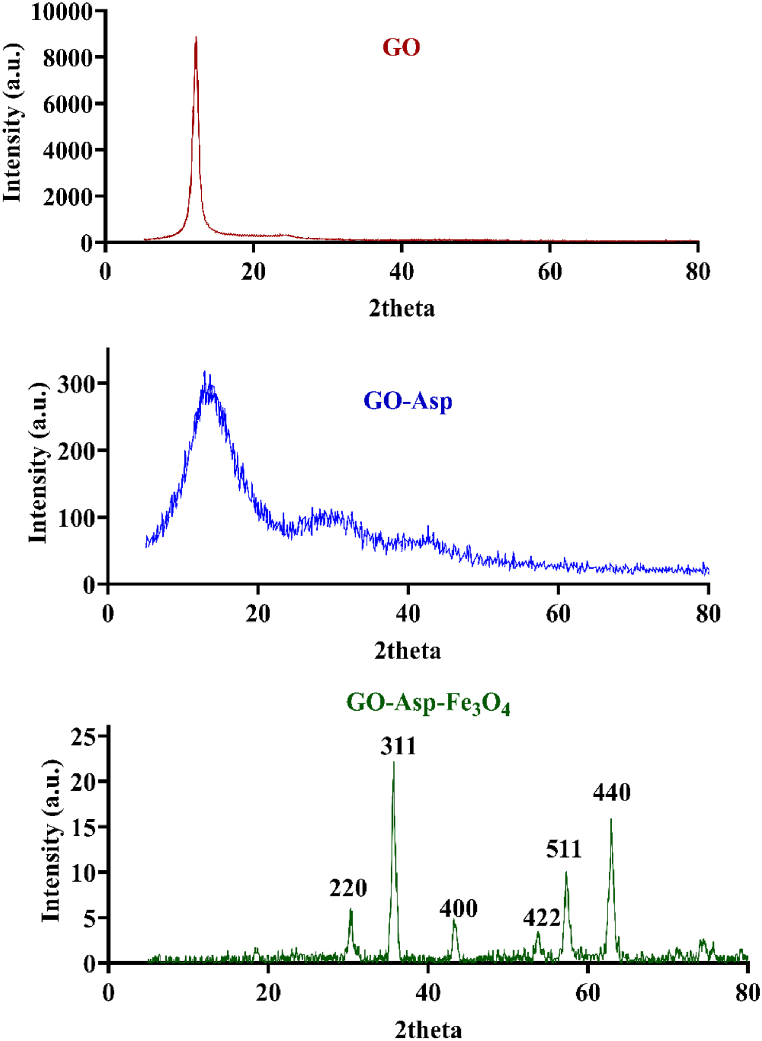
XRD patterns of GO, GO-Asp, GO-Asp-Fe_3_O_4_.

Crystalline structure and polymorphism were examined by XRD (Fig. 4). GO is not fully crystalline; it has crystalline and amorphous parts. This means that while it doesn't have a perfect crystal structure, it still exhibits some degree of crystallinity, which can be detected and analyzed using XRD. The oxygen-containing functional groups disrupt the perfect graphene lattice, forming a mixture of crystalline and amorphous regions. The degree of crystallinity depends on the extent of oxidation. GO exhibits a sharp diffraction peak at a 2θ value of 11.06°, which is attributed to the (002) reflection [27,28]. GO modification with Asp led to decreased crystallinity, as evidenced by the reduction in peak intensity [29]. This is consistent with other studies where functionalization with various molecules, such as amines or thiols, has resulted in reduced peak intensity, indicating a decrease in crystallinity due to the disruption of the GO lattice [29]. In addition, GO functionalization can change the stacking order and interlayer spacing, which can be analyzed by the broadening and shift in 002 plane peak. Upon chemical functionalization with L-Asp, this peak shifts to a 2θ value of 13.04°, indicating that the d-spacing decreased from 8 Å to 6 Å due to the modification of GO with Asp. This change suggests that inter-layer interactions within the GO structure are triggered by the presence of Asp [30]. When synthesizing in-situ magnetic nanoparticles on GO, several polymorphic forms of the magnetic nanoparticles including magnetite, maghemite, and hematite can be produced, depending on the synthesis conditions such as temperature, pH, and precursor materials [31]. However, each polymorphism has a specific crystal plane that can be detected via the XRD technique. In the present study, XRD pattern of the GO-Asp-Fe_3_O_4_ nanocomposite exhibits six characteristic peaks of Fe_3_O_4_ at around 2θ = 30.42, 35.72, 43.54, 53.92, 57.36, and 62.96, corresponding to the crystallographic indices (220), (311), (400), (422), (511), and (440), respectively. These peaks closely match the positions and intensities of magnetite as per the JCPDS card no.75-1610 [32]. Moreover, the disappearance of the (002) reflection peak of GO is attributed to the inability of the GO sheets to form crystalline structures once they are coated with the magnetic nanoparticles.

The crystallite size of the synthesized materials was also calculated using the Debye−Scherrer equation (D = 0.94 λ/(β cos θ), where λ represents the X-ray wavelength (0.15404 nm), β indicates the full width at half-maximum, and θ denotes the diffraction angle). Based on this equation, the crystallite size was 8.35 nm for GO nanoparticles and 1.13 nm for the GO-Asp composites. The decrease in average crystallite size for GO-Asp compared to GO suggests a reduction in graphene regions and the formation of more lateral defects, resulting in increased surface area and reactivity, which can enhance properties like chemical reactivity and catalytic activity [33]. Additionally, the average crystallite size of Fe_3_O_4_ nanoparticles was approximately 17.2 ± 1.3 nm, highlighting their nanoscale dimensions.

**Fig. 5:**
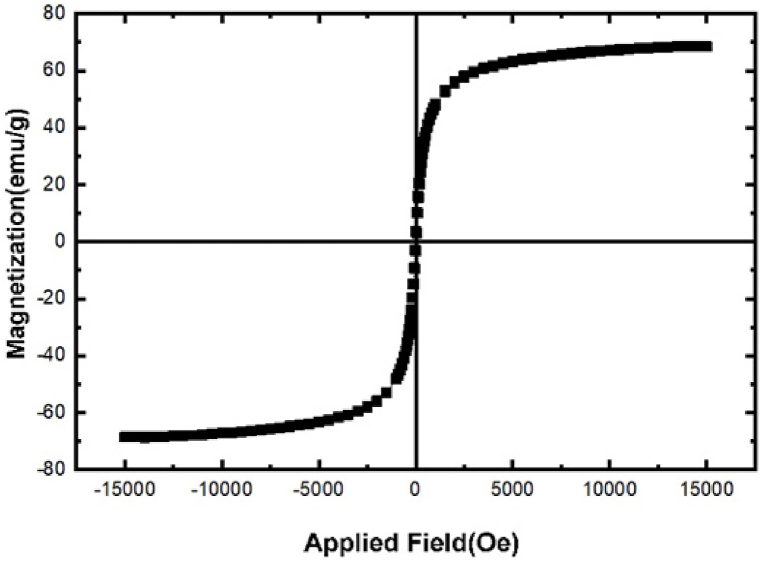
Magnetization curve for GO-Asp-Fe_3_O_4_.

The magnetization curve of GO-Asp-Fe_3_O_4_ (as shown in Fig. 5) demonstrates a specific saturation magnetization (Ms) of 70 emu. This characteristic high magnetic property of GO-Asp- Fe_3_O_4_ is desirable for various applications, including industrial enzyme immobilization, magnetic resonance imaging, and targeted delivery. Additionally, the absence of coercivity indicates the superparamagnetic nature of the immobilized Fe_3_O_4_ nanoparticles.

#### 3.1.1 Morphology and zeta potential measurement

**Fig. 6:**
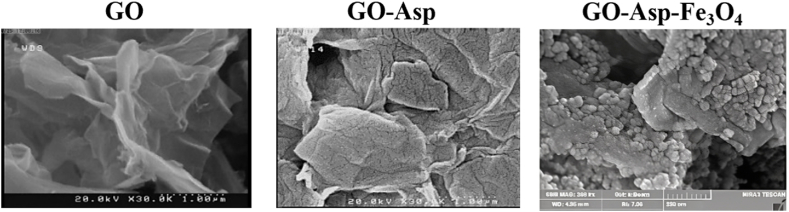
FE-SEM images of GO, GO-Asp, and GO-Asp-Fe_3_O_4_.

**Fig. 7:**
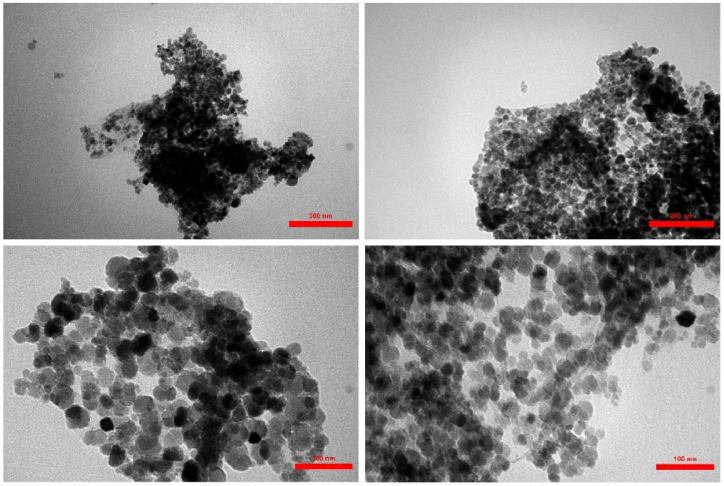
TEM images of GO-Asp-Fe_3_O_4_.

To investigate the surface morphological changes of GO upon functionalization and magnetization, electron microscopy was applied. The FE-SEM micrograph (Fig. 6) shows that GO has a well-exfoliated sheet-like morphology with curled edges. Following L-Asp immobilization, the GO nanosheets exhibited a rougher surface than unmodified GO, with an increased wrinkle depth. Furthermore, the transparency of the graphene layers was notably reduced, indicating a successful GO modification. There was a noticeable variation in morphology between GO-Asp and GO-Asp-Fe_3_O_4_. The GO-Asp-Fe_3_O_4_ nanosheets showed a uniform covering of spherical Fe_3_O_4_ nanoparticles with an average size of 21 ± 5.7 nm on the GO-Asp nanosheets. Consistent with these findings, TEM images of GO-Asp-Fe_3_O_4_ depicted the presence of Fe_3_O_4_ nanoparticles on the graphene layers in the form of spherical particles (Fig. 7). These particles exhibited a uniform distribution and an average size of 24 ± 8 nm which is in agreement with the particle size obtained from SEM microscopy and XRD analysis.

The DLS measurement was utilized to examine the average hydrodynamic particle size distribution of the prepared samples. The average hydrodynamic diameter of GO-Asp was 101 nm (PDI: 0.1), which grows to 160 nm (PDI: 0.12) after Fe_3_O_4_ immobilization, indicating the presence of the Fe_3_O_4_ nanoparticles on the graphene nanosheets. After the immobilization of ASNase, the size further increased to 179 nm (PDI: 0.14). Moreover, the surface zeta potential changed with the alterations in the GO surface. The pristine GO sheets, due to the presence of the negatively charged oxygen-containing groups exhibited a zeta potential of −48.7 ± 2.3 mV. The GO-Asp surface showed a higher negative charge (−61.4 ± 3.1 mV), potentially due to the incorporation of two carboxylate groups from each L-Asp molecule onto the GO surface. However, after magnetization, the presence of Fe_3_O_4_ reduced the overall zeta potential of GO nanosheets to −54.8 ± 2.3 % mV. After the immobilization of ASNase, the zeta potential significantly dropped to −9.8 ± 0.8 mV, proving successful attachment of the enzyme. The covalent bonding of ASNase with carboxylic groups on the GO surface led to a substantial decrease in the negative charges of the GO. The modified zeta potential suggests improved interaction with biological membranes and other biomolecules, potentially increasing the efficacy of ASNase in therapeutic contexts.

### 3.2 Optimization of ASNase immobilization on GO-Asp-Fe_3_O_4_ nanocomposite

**Fig. 8:**
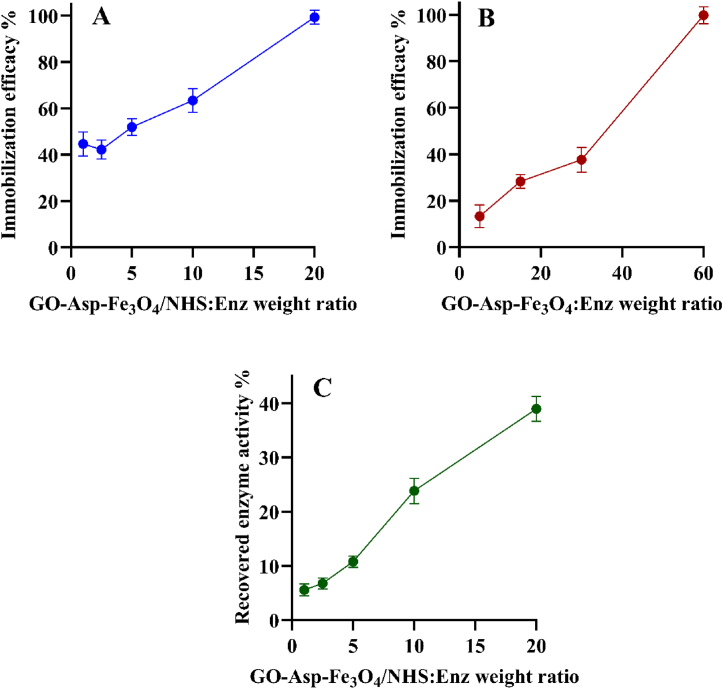
Immobilization efficiency versus GO-Asp-Fe_3_O_4_:Enz weight ratio for A) chemical and B) physical L-asparaginase immobilization. C) Recovered enzyme activity versus GO-Asp-Fe_3_O_4_:Enz weight ratio for covalently immobilized L-asparaginase. Data represent mean ± SD, n = 3.

The GO-Asp-Fe_3_O_4_ composite was investigated as a nano-support for the covalent or non-covalent immobilization of ASNase. For covalent attachment of the enzyme, the carboxylic groups of magnetic GO-Asp were first functionalized with EDC/NHS and then reacted with ASNase lysine amine groups via the nucleophilic substitution. The covalent linkage between the amine and carboxylic acid groups prevents rapid leaching of the enzyme from the support [34]. The efficacy of the protein immobilization was then assayed by the Bradford method. It was expected that the carrier-to-enzyme weight ratio and GO functional group could affect the immobilization efficacy. According to Fig. 8-A, for the EDC/NHS activated GO-Asp-Fe_3_O_4_ at a fixed ASNase concentration, with an increase in the carrier-to-enzyme weight ratio from 1 to 20, the conjugation degree increased from 44.7 % ± 5.2 %–99.2 % ± 2.9 %. The same trend was also observed in the case of the physical adsorption method (Fig. 8-B). However, for the non-activated GO-Asp, the complete immobilization of ASNase was achieved at a carrier-to-enzyme weight ratio of 60, and at a weight ratio of 20 only about 34 % of the input ASNase was attached. Proteins could be absorbed on GO non-covalently through hydrophobic, electrostatic, and π-π stacking, and van der Waals forces interactions. Herein, the L-Asp functionalization and magnetization of GO reduced the hydrophobicity and the possibility of physical interaction of ASNase with graphene sheets which could be a contributing factor to the reduced ASNase loading on GO-Asp. Alam et al. described the maximum efficiency of ASNase immobilization on APTES-modified magnetic nanoparticles was 62 % [35]. In comparison, for Fe_3_O_4_-chitosan and magnetic Fe_3_O_4_@MCM-41, the immobilization efficacy was 73.2 % and 63 %, respectively. These findings suggest that activated GO-Asp-Fe_3_O_4_ nanocomposite have a high immobilization yield making them a promising support for immobilizing L-ASNase. Examination of enzyme activity for EDC/NHS-activated GO-Asp-Fe_3_O_4_ revealed that as the carrier-to-enzyme weight ratio rose from 1 to 20, enzyme activity increased from 5.6 % ± 1.1 %–38.9 % ± 2.3 %, demonstrating successful enzyme immobilization (Fig. 8-C). Research showed that the enzyme's catalytic activity decreased slightly after modification [36]. In the current research, the recovered enzyme activity was 38.9 % ± 2.3 % when the initial enzyme was fully immobilized likely due to steric hindrance of the matrix leading to decreased substrate accessibility [37]. Based on these results, GO-Asp-Fe_3_O_4_/ASNase chemically prepared at the carrier-to-enzyme weight ratio of 20 was selected for the rest of the experiments.

### 3.3 Determination of kinetic parameters

**Fig. 9:**
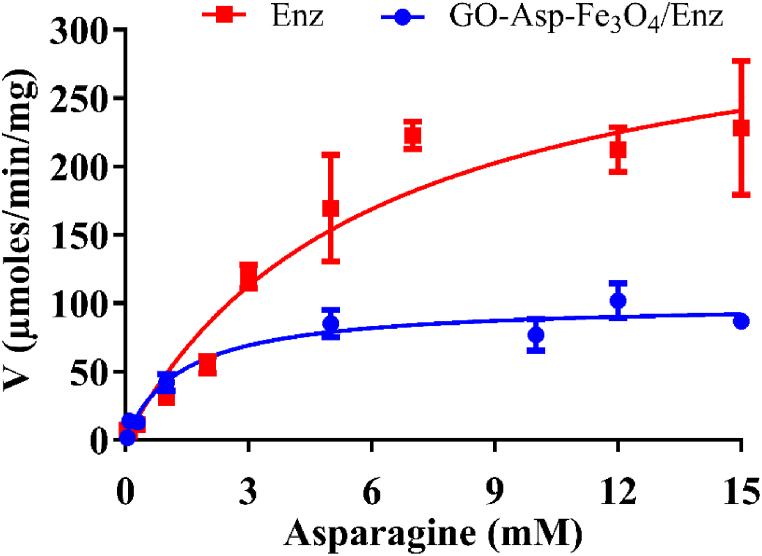
Plots of the reaction rate (V) of native and immobilized L-asparaginase on GO-Asp-Fe_3_O_4_ versus L-asparagine concentration (0.05–15 mM). The data is presented as mean ± standard deviation, with a sample size of n = 3 and fitted to the Michaelis-Menten equation.

The *K*_m_ and *V*_max_ values, kinetic parameters, of the free and immobilized enzyme, were evaluated using substrate L-asparagine (Fig. 9) and summarized in Table 1. The *V*_max_ parameter represents the maximum rate of reaction when substrate molecules fill every active site of the enzyme. GO-Asp-Fe_3_O_4_/Enz exhibited a significant decrease in *V*_max_ value (100 ± 5 U/mg) compared to the free enzyme (337 ± 35 U/mg). This reduction in activity could be attributed to limitations in substrate diffusion, hindrance caused by steric factors of the support material, or a decrease in the flexibility of the enzyme which is essential for the enzyme-substrate complex formation [38]. On the other hand, the *K*_m_ value of the enzyme decreased approximately 4.4-fold after immobilization, indicating the higher affinity of the ASNase towards the substrate. Similar findings were documented for immobilized ASNase over carbon xerogels and magnetic chitosan.

**Table 1:** The Michaelis-Menten constants for free and immobilized L-asparaginase on GO-Asp-Fe_3_O_4_.

Sample	K_m_ (mM) ± SD	V_max_ (U/mg) ± SD
Enz	6 ± 1	337 ± 35
GO-Asp-Fe_3_O_4_/Enz	1 ± 0	100 ± 5

### 3.4 Dispersibility

**Fig. 10:**
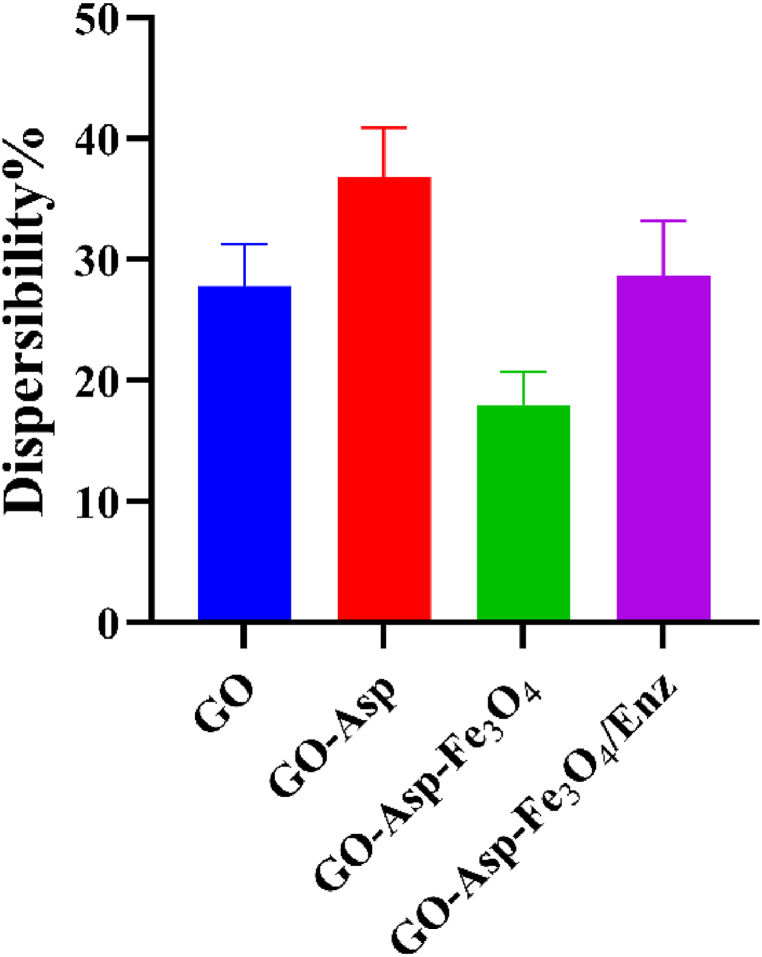
Aqeuous dispersibility of GO, GO-Asp, GO-Asp-Fe_3_O_4_, and GO-Asp-Fe_3_O_4_/ENZ.

Surface modifications impact the dispersion characteristics of GO, which is crucial for the best performance of the composite in applications such as catalysis and drug delivery. According to Fig. 10, modification of GO with Asp enhanced the GO dispersion possibly due to increased surface hydrophilicity [39]. On the other hand, the dispersibility was reduced to 18.3 % ± 2.7 % for Go-Asp-Fe_3_O_4_. Because Fe_3_O_4_ nanoparticles have a larger density than unmodified GO, they can settle in suspension more quickly after centrifugation in composite particles. In addition, the Fe_3_O_4_ modification of GO decreases dispersibility by increasing particle size and reducing the surface area exposed to the solvent. After immobilization of the enzyme, the dispersibility raised to 28.7 % ± 4.5 %. This increase in dispersibility can be attributed to the enhanced hydrophilicity that may arise from this post-modification. The enzyme could expose more hydrophilic functional groups or stabilize the dispersion by creating a more favorable microenvironment for interaction with the solvent.

### 3.5 pH and thermal stability

**Fig. 11:**
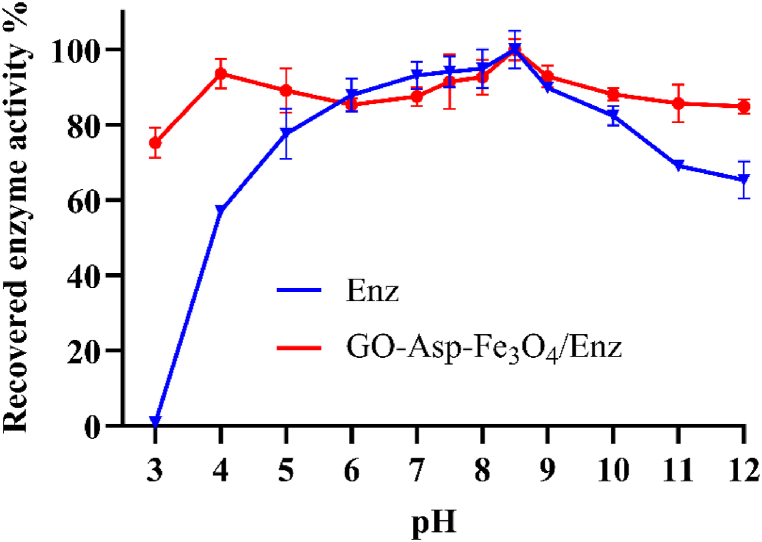
Effect of pH on free and immobilized L-asparaginase recovered enzyme activity in a pH range of 3–12.

pH and thermal stability are two important characteristics for industrial applications and storage stability of biomedical enzymes. Fig. 11 demonstrates the effect of immobilization on the pH stability of ASNase in comparison to the free enzyme. Both the free and immobilized ASNase enzymes exhibited over 80 % of their catalytic activity within the pH range of 5–9. However, the activity of the free enzyme sharply decreased in extreme pH conditions, with a complete loss of activity at pH 3. Interestingly, the immobilization of ASNase on GO-Asp-Fe_3_O_4_ promoted pH stability. The activity recovery of GO was 75 % ± 4 % and 84 % ± 2 % in pH values of 3 and 12, respectively. The improved stability of ASNase through covalent attachment could be attributed to reduced enzyme leaching and protein deformation under varying pH conditions. Furthermore, the active site of the native ASNase is susceptible to elevated concentrations of H^+^ ions. Hence, the protective effect of GO-Asp-Fe_3_O_4_ could preserve the enzyme's structure and activity under acidic pH values.

**Fig. 12:**
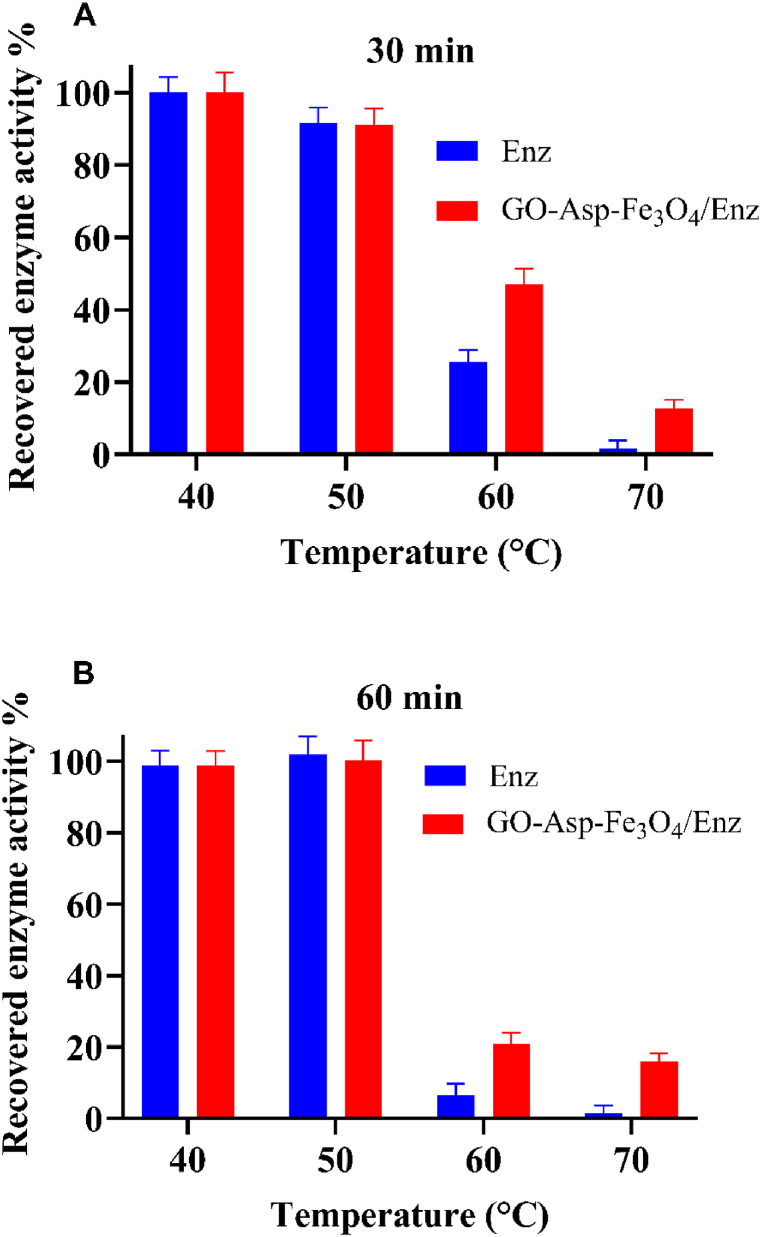
Effect of temperature on free and immobilized L-asparaginase after A)30 min and B)60 min incubation in a range of 40–70 °C.

The thermal stability of free and immobilized ASNase was explored within a temperature range of 40–70 °C. According to Fig. 12-A and 12-B, ASNase and GO-Asp-Fe_3_O_4_/Enz remained stable for up to 1 h at 50 °C. Elevating the temperature to 60 °C led to a significant decrease in the enzymatic activity of free ASNase, with a residual enzyme activity of 24 % ± 3 % and 6 % ± 2 % after 30 and 60 min of incubation, respectively. In contrast, the GO-Asp-Fe_3_O_4_/Enz exhibited higher residual enzyme activity of 47 % ± 3 % and 21 % ± 3 % within the same incubation times, respectively. At 70 °C, the free ASNase lost almost all of its enzyme activity, while the immobilized ASNase retained 16 % of its original activity. According to these findings, the recovered enzyme activity of the immobilized ASNase was two-to eight-fold higher than that of the free enzyme. Previous research has also documented the elevated thermal stability of ASNase through immobilization on calcium alginate-gelatin composites and carboxymethyl cellulose matrix [40]. The enhanced thermal tolerance of the immobilized enzyme could be attributed to a reduction in enzyme chain flexibility and an elevation in the activation energy required for enzyme deformation.

### 3.6 Reusability

**Fig. 13:**
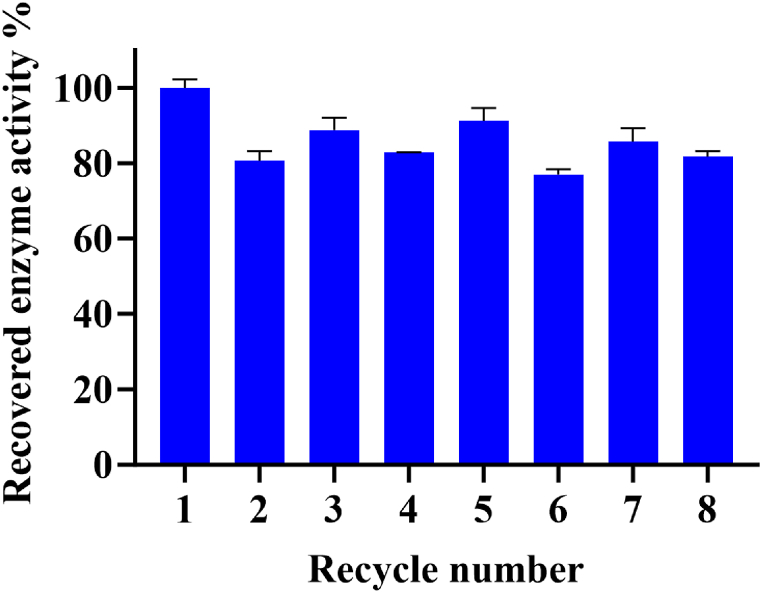
Profile of reusability of immobilized L-asparaginase on GO-Asp-Fe_3_O_4_.

The potential applications of enzymes in large-scale or industrial settings greatly rely on their reusability. Enzyme immobilization has shown to be a promise in facilitating their recovery across multiple cycles, thereby reducing overall process costs. In the present study, we noticed a slight decrease in enzyme activity for GO-Asp-Fe_3_O_4_/Enz in the second cycle, potentially due to the leaching of physically absorbed enzymes (Fig. 13). However, in the subsequent six consecutive cycles, the recovered enzyme activity remained consistently high at 80 %. This suggests that the covalent attachment of ASNase to GO nanosheets can prevent protein leaching and conformational changes during repeated use. The notable reusability aligns with the positive impact of covalent immobilization on thermal stability as reported in section 3.4. In comparison with non-magnetic GO-Asp [20], the covalent immobilization of ASNase on the novel GO-Asp-Fe_3_O_4_ led to an approximately twofold increase in the final recovered activity, underscoring the high potential applicability of magnetized GO-Asp-Fe_3_O_4_ in the food and biotechnology industries. This result is in good consistency with other enzymes immobilized on magnetic GO, for example, the reported recovered activity after 8 cycles is 68 % for laccase [41], 88 % for lactase [42], 79 % for ficin [43], and 80 % for b-xylosidase [44].

## 4 Conclusion

In this study, our objective was to enhance the stability and reusability of ASNase, a crucial enzyme for applications in biotechnology and the food industry. We achieved this goal by developing a novel biological nano-biocatalyst through the covalent immobilization of ASNase onto hybrid nanomaterials composed of Asp-functionalized GO and Fe_3_O_4_ nanoparticles. Through careful optimization of the carrier-to-protein weight ratio, we achieved complete enzyme immobilization. Our results indicate that the immobilized ASNase exhibits superior thermal stability compared to its free counterpart. Furthermore, the GO-Asp-Fe_3_O_4_ nano-support significantly enhances enzyme catalytic activity, particularly at extreme pH levels. Notably, this immobilization platform offers high enzyme recovery across various reaction media, enhanced substrate affinity, and impressive reusability. These findings position GO-Asp-Fe_3_O_4_ as a highly favorable option for enzyme immobilization platforms in a range of industries.

## CRediT authorship contribution statement

**Maryam Monajati:** Writing – review & editing, Writing – original draft, Methodology. **Nasim Ariafar:** Writing – review & editing, Investigation. **Mehdi Abedi:** Writing – review & editing, Investigation. **Sedigheh Borandeh:** Writing – review & editing, Project administration, Conceptualization. **Ali Mohammad Tamaddon:** Writing – review & editing, Project administration, Conceptualization.

## Ethical approval

This study was approved by the Research Ethics Committee of Shiraz University of Medical Sciences (Ethical Code: IR.SUMS.REC.1398.031).

## Consent to participate

Not applicable.

## Consent to publication

Not applicable.

## Availability of data and materials

The datasets are available from the corresponding author on reasonable request.

## Funding

This study was funded by a grant from Shiraz University of Medical Sciences (SUMS), Deputy of Research and Technology, research project no. 97-01-81-18265.

## Declaration of competing interest

The authors declare the following financial interests/personal relationships which may be considered as potential competing interests:Alimohammad Tamaddon reports financial support was provided by 10.13039/501100004320Shiraz University of Medical Sciences. If there are other authors, they declare that they have no known competing financial interests or personal relationships that could have appeared to influence the work reported in this paper.
